# Treatment of biochemical recurrence after primary therapy with curative intent

**DOI:** 10.1097/MOU.0000000000001312

**Published:** 2025-07-07

**Authors:** Navid Roessler, Marcin Miszczyk, Nadja Strewinsky, Paweł Rajwa, Shahrokh F. Shariat

**Affiliations:** a Department of Urology, Comprehensive Cancer Center, Medical University of Vienna, Vienna, Austria; b Department of Urology, Medical University Center Hamburg-Eppendorf, Hamburg, Germany; c Collegium Medicum, Faculty of Medicine, WSB University, Dąbrowa Górnicza, Poland; d Department of Oncology, Hematology and Bone Marrow Transplantation with Section Pneumology, University Center Hamburg-Eppendorf, Hamburg, Germany; e Department of Urology, Centre of Postgraduate Medical Education, Warsaw, Poland; f Division of Surgery and Interventional Sciences, University College London, London, UK; g Department of Urology, University of Texas Southwestern, Dallas, Texas, USA; h Department of Urology, Second Faculty of Medicine, Charles University, Prague, Czech Republic; i Hourani Center for Applied Scientific Research, Al-Ahliyya Amman University, Amman, Jordan; j Karl Landsteiner Institute of Urology and Andrology, Vienna, Austria; k Research Center for Evidence Medicine, Urology Department, Tabriz University of Medical Sciences, Tabriz, Iran

**Keywords:** biochemical recurrence, prostate cancer, PSMA-PET, salvage therapy

## Abstract

**Purpose of review:**

We aimed to summarize the recent advancements in management of biochemical recurrence (BCR) after primary curative therapy for prostate cancer (PCa), and the role of advanced imaging technologies in guiding and improving treatment decisions.

**Recent findings:**

Recent studies have reshaped the approach to managing BCR after primary treatment for PCa. A key shift is the preference for early salvage radiotherapy (sRT), which has proven to offer comparable or even superior outcomes to immediate adjuvant therapy when closely monitored for progression. PSA kinetics (PSA doubling time) continue to guide treatment decisions, together with the time to PSA rise, Gleason Grade of the original tumor, and PSMA-PET imaging at the time of recurrence. While PSMA-PET significantly enhances the precision of recurrence detection, its sensitivity for smaller pelvic lymph node metastases remains limited, underscoring the need for careful consideration of all factors together to develop a risk-based consulting for all individualized treatment plan integrating patient wishes and health.

**Summary:**

Recent studies underscore the efficacy of early sRT in managing BCR, with PSA kinetics and ISUP score as a crucial factor in guiding treatment decisions. Furthermore, the integration of PSMA-PET imaging has improved the precision of recurrence detection, facilitating more tailored and effective treatment strategies for patients with BCR. We are finally entering the age of personalized, risk-based, patient-centred case delivery, where treatment of the primary tumor with curative intent is offered to patients with BCR.

## INTRODUCTION

Biochemical recurrence (BCR) affects up to 50% of patients treated with radiotherapy (RT) or radical prostatectomy (RP) for clinically localized prostate cancer (PCa) with curative intent [1]. BCR is a critical event in PCa, as it signals the potential for disease progression and the need for timely intervention to prevent metastatic spread. Managing BCR often requires a multidisciplinary team approach, balancing between the risk of overtreatment, with its associated adverse events, and missing the deep sustained disease control. Recent high-quality evidence addressed many aspects of timing, extent, and intensity of salvage RT and systemic therapy, as well as the ongoing advancements in the field of modern imaging (i.e. PSMA-PET). In this review, we focused on synthesizing data from contemporary trials providing practice-changing evidence. 

**Box 1:**
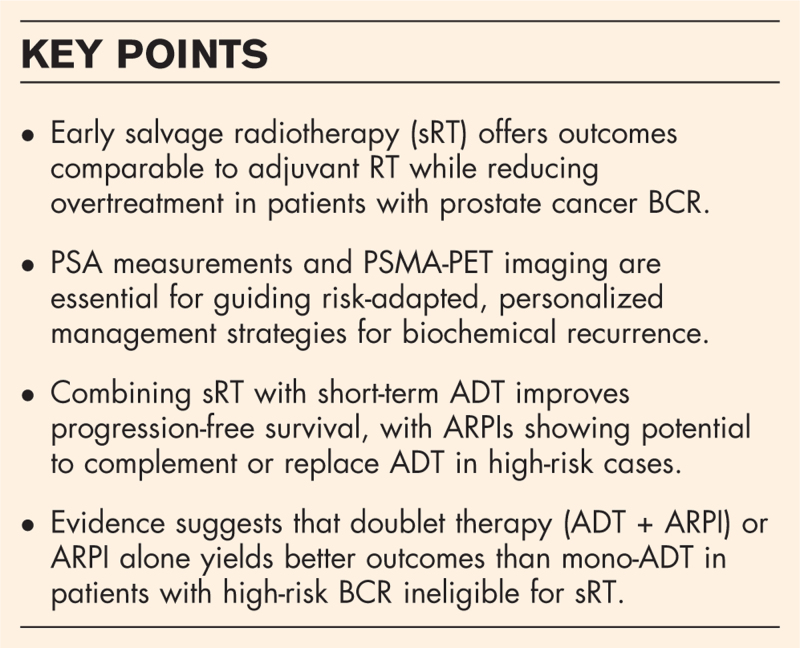
no caption available

## BIOCHEMICAL RECURRENCE: DEFINITION AND IMAGING

The definition of BCR depends on the primary treatment modality. After RP, a PSA level rising above 0.2 ng/ml is a widely accepted as the threshold for BCR, although a PSA level exceeding 0.4 ng/ml has been shown to have better discriminatory value for predicting the development of metastases [2–4]. However, there is no definitive PSA threshold for treatment failure, and any PSA rise should be interpreted according to the EAU BCR risk stratification [5], which has been validated and offers a comprehensive assessment of recurrence risk [6]. After primary RT, a PSA increase ≫2 ng/ml above posttreatment nadir predicts clinical failure with high accuracy [4]. However, current guidelines emphasize that PSA level alone should not be considered as definitive marker of treatment failure, as it must be interpreted alongside other clinical parameters to accurately assess the risk of distant failure [7]. In this context, PSA bounce refers to a temporary rise in PSA levels followed by a spontaneous decline, sometimes observed after RT with high fraction doses or brachytherapy for localized PCa, and should be distinguished from true BCR to avoid unnecessary and meaningful interventions [8]. While ultrasensitive PSA testing can detect BCR and inform salvage treatment decisions at concentrations of ≤0.1 ng/ml post-RP [9], the clinical relevance of low-detectable PSA values is not clear, and an agreement regarding the optimal PSA threshold for initiating therapy has not been reached. It is often pointed out that a single PSA value is insufficient for risk assessment; PSA doubling time (PSA-DT) can also be used as a prognostic marker, with shorter PSA-DT indicating higher risk of progression [7,10]. In advanced or recurrent PCa, PSA kinetics, including doubling time, are well established prognostic markers [11], but their interpretation should be context-dependent. Pretreatment factors such as tumour stage, ISUP score, and PSA levels influence the individual risk of recurrence [7]. Genomic testing with DECIPHER could complement clinical models in assessing the risk of BCR after primary treatment; however, data from prospective trials is missing [12]. Upon diagnosis of BCR, both the European Association of Urology (EAU) and the National Comprehensive Cancer Network (NCCN) suggest that selected low-risk patients, defined by a PSA-Doubling Time (PSA-DT) >12 months and ISUP grade 1–3, may be considered candidates for deferring therapy [13,14].

Prostate-specific membrane antigen positron emission tomography (PSMA-PET) has significantly improved our ability to detect lesions underlying a BCR, facilitating development of personalized treatment strategies [15]. Conventional imaging modalities, such as CT and bone scans, demonstrate limited sensitivity in patients with low PSA values [16–18]. In contrast, PSMA-PET can identify metastatic lesions at a PSA level of as low as <0.5 ng/ml [19], providing superior accuracy [20–22] that leads to better-informed decision-making [23]. However, there are limitations – preliminary results of the ongoing ‘PEACE-V-STORM’ trial, as presented at the ESTRO 2024 congress, show that omitting elective pelvic lymph node irradiation and treating only PSMA-PET visible lesions in patients with pelvic nodal oligo-recurrence leads to a significantly worse 3-year biochemical relapse-free survival (47% vs. 69%, *P* = 0.01) [24]. This is in line with the findings of Hope *et al.*, who showed that in the context of primary treatment, despite high specificity (95%), PSMA-PET has limited sensitivity (40%) for detecting very small pelvic lymph node metastases. Considering that salvage RT (sRT) is a very effective treatment strategy in patients with low PSA levels, typically lower than the thresholds associated with positive findings on PSMA-PET, it is not recommended to postpone sRT until PCa lesions can be identified by PSMA-PET, as doing so may cause PSA to rise and subsequently decrease the chance for cure [25]. It has been shown that patients with negative PSMA PET/CT results who subsequently undergo sRT demonstrate a high treatment response, while those who do not receive treatment experience a continued increase in PSA levels [26]. That said, emerging evidence suggests that PSMA-PET prior to salvage therapy may improve biochemical control through enhanced treatment personalization [27]. However, randomized controlled trials (RCTs) are required to confirm whether this benefit reflects a causal relationship or stems from residual selection bias and confounding factors.

## SALVAGE THERAPY AFTER DEFINITIVE TREATMENT

The ‘RAVES’ trial, a phase III RCT, compared the risk of biochemical progression-free survival (BPFS) between patients with clinically nonmetastatic PCa, who had primary RP (*n* = 333) and postoperative PSA levels ≤0.1 ng/ml, and received either adjuvant RT (*n* = 166) or early sRT at BCR (*n* = 167) [28]. There was no significant difference between the groups [5-year BPFS: 86% vs. 87%; hazard ratio (HR): 1.12; 95% confidence interval (CI): 0.65–1.9; *P* = 0.15] [28]. Similar findings were described in another phase III RCT called ‘RADICALS-RT’ [29^▪▪^]. Patients with nonmetastatic PCa treated with primary RP (*n* = 1.396) and postoperative PSA levels ≤0.2 ng/ml received either adjuvant RT (*n* = 697) or early sRT at BCR (*n* = 699) [29^▪▪^]. There was no significant difference between the groups (5-year BPFS: 85% vs. 88%; HR 1.1; 95% CI 0.81–1.49; *P* = 0.56) [29^▪▪^]. Finally, the ‘GETUG-AFU 17’ phase III RCT compared event-free survival (EFS), a composite endpoint comprising clinical, biochemical, and radiologic events, in patients with clinically nonmetastatic PCa, who had primary RP (*n* = 424) and postoperative PSA levels ≤0.1 ng/ml [30]. The patients received either adjuvant RT (*n* = 212) or early sRT at BCR (*n* = 212) [30]. There was no significant difference in EFS between groups (5-year EFS: 92% vs. 90%; HR: 0.81; 95% CI: 0.48–1.36; *P* = 0.42) [30]. Those findings show, that in contemporary era of wide access to sensitive PSA testing, early sRT is a preferred approach, allowing to spare or delay unnecessary interventions in many patients. This conclusion was further reinforced by a subsequent meta-analysis that did not show any significant improvement in EFS with adjuvant RT (5-year EFS 89% vs. 88%; HR: 0.95; 95% CI 0.75–1.21; *P* = 0.70) based on pooled, harmonized, and updated data of 2153 patients treated within the frame of these three RCTs [31].

Remaining questions that needed to be addressed included the concomitant use of hormone therapy (HT), and the extent of the irradiation; that is – whether sRT should include elective pelvic lymph node irradiation. In the ‘SSPORT’ trial, a three-arm RCT, patients were randomized to receive sRT to the prostate alone (*n* = 564), sRT to the prostate combined with short-term HT (*n* = 578), or sRT to the prostate with a simultaneous pelvic lymph node irradiation and short-term HT (*n* = 574) [32^▪▪^]. The addition of HT was associated with a significant improvement in freedom from progression (FFP), a composite endpoint comprising clinical and biochemical failure [32^▪▪^]. The five-year FFP was 81.3% for patients receiving sRT and HT, compared to 70.9% for prostate sRT alone (HR 0.60; 97.5% CI: 0.47–0.77; *P* ≤ 0.001) [32^▪▪^]. The 5-year FFP was also improved in patients receiving HT and sRT to the prostate and elective pelvic irradiation, compared to HT combined with sRT to the prostate only (87.4% vs. 81.3%; HR 0.82; 97.5% CI: 0.63–1.07; *P* = 0.048), providing invaluable data on the impact of extent and intensity of sRT on the oncologic outcomes of the patients [32^▪▪^]. The aspect of concomitant short-term was also evaluated in the ‘GETUG-AFU 16’ RCT [33^▪^]. Patients were randomized to receive six months of concomitant goserelin with sRT (*n* = 369), or sRT alone (*n* = 374) [33^▪^]. The progression-free survival (PFS), inclusive of clinical and biochemical failure, was significantly improved in patients receiving HT (10-year PFS 64% vs. 49%; HR 0.54; 95% CI: 0.43–0.68; *P* < 0.0001) [33^▪^], conclusively establishing the important role of short-term ADT in mitigating or deferring progression events in patients undergoing sRT. However, recent data from the ‘RADICALS-HD’ trial, comparing 24 months of long-course ADT (*n* = 762) with 6 months of short-course ADT (*n* = 761), demonstrated that long-course ADT improved 10-year metastasis-free survival (MFS) to 78.1% vs. 71.9% (HR 0.77; 95% CI: 0.61–0.97; *P* = 0.029), suggesting a benefit for extending the duration of ADT [34].

Treatment options for patients with BCR after primary RT include HT, local salvage procedures, and a watchful waiting approach. Local treatments, including salvage RP, brachytherapy, repeated RT, high-intensity focused ultrasound, and cryosurgical ablation should be considered only for selected patients with biopsy-proven local recurrence, ideally within the frame of clinical trials or well designed prospective registries at expert centres [7]. The ‘MASTER’ systematic review and meta-analysis did not identify statistically significant differences in 5-year recurrence-free survival across salvage modalities [35]; however, there was vast heterogeneity with regard to endpoint definition and data quality, ranging from case series to prospective studies, and toxicity assessment methods were inconsistent, often relying on nonstandardized descriptive criteria [36]. ADT-based HT remains a standard of care, but also a matter of debate, as there is conflicting evidence regarding its effectiveness [37,38]. Finally, the recent RCT called ‘EMBARK’ assessed the efficacy and safety of ADT (leuprolide) plus enzalutamide (*n* = 355) and enzalutamide monotherapy (n = 355) compared to ADT alone (*n* = 358) in PCa patients with high-risk BCR after RT, or BCR after RP who were not candidates for sRT [39^▪▪^]. The 5-year metastasis-free survival (MFS) rates were 87.3% (95% CI: 83–90.6) for enzalutamide plus ADT, 71.4% (95% CI: 65.7–76.3) for ADT alone, and 80% (95% CI: 75–84.1) for enzalutamide monotherapy [39^▪▪^]. Both enzalutamide plus ADT (HR: 0.42; 95% CI: 0.30–0.61; *P* < 0.001) as well as enzalutamide monotherapy (HR: 0.63; 95% CI: 0.46–0.87; *P* = 0.005) significantly improved MFS compared to ADT alone [39^▪▪^]. This indicates a possible shift in the paradigm; it is likely that ADT will be replaced by ARPIs as the backbone of systemic therapy, as the latter appear as a more potent drug. ARPI monotherapy also becomes an option for patients wishing to reduce sexual toxicity; however, with an offset of a significant increase in other domains, such as breast pain and gynecomastia. Considering the ultimately nondefinitive intent of this therapy, EMBARK-like HT should be reserved for patients at highest risk of failure, with disease harbouring aggressive features, who are not candidates for standard-of-care sRT [7].

## CONCLUSION

The management of BCR in PCa is increasingly shifting towards personalized treatment strategies, driven by advancements in imaging and systemic therapies. PSMA-PET imaging enables earlier and more accurate recurrence detection, allowing for more tailored interventions, yet should not delay the decision for salvage intervention. While the role of sRT, particularly in context of pelvic lymph node irradiation, is still being explored, novel systemic treatments like enzalutamide are emerging as promising alternatives to traditional ADT. Moving forward, the integration of advanced imaging, genomic data, and novel treatments will be crucial in optimizing outcomes while minimizing overtreatment in BCR management.

## Acknowledgements

*None.*

### Financial support and sponsorship

*Marcin Miszczyk was supported by the European Urological Scholarship Programme (EUSP) Scholarship of the European Association of Urology (EAU).*

### Conflicts of interest

*There are no conflicts of interest.*

## REFERENCES AND RECOMMENDED READING

Papers of particular interest, published within the annual period of review, have been highlighted as:

▪ of special interest
▪▪ of outstanding interest
